# Scopus-based bibliometric analysis of research trends in silage feed and its impact on rumen fermentation in ruminants

**DOI:** 10.14202/vetworld.2025.1972-1990

**Published:** 2025-07-22

**Authors:** Tri Rachmanto Prihambodo, Randi Mulianda, Wulandari Wulandari, Santika Anggrahini, Novia Qomariyah, Andi Ella, Erna Winarti, Yenni Yusriani, Suyatno Suyatno, Jhon Firison, Deni Fitra, Anwar Efendi Harahap, Dinda Ayu Permata Sari, Taufik Hidayat, Anuraga Jayanegara

**Affiliations:** 1 Department of Animal Science, Faculty of Animal Science, Jenderal Soedirman University, Purwokerto, Indonesia; 2 Research Center for Animal Husbandry, National Research and Innovation Agency (BRIN), Bogor, Indonesia; 3 Animal Science Study Program, Faculty of Agriculture and Animal Science, Sultan Syarif Kasim State Islamic University Riau, Pekanbaru 28293, Indonesia; 4 Department of Animal Science, Faculty of Animal Sciences, Diponegoro University, Semarang, Indonesia; 5 Research Center for Hortikultura, National Research and Innovation Agency (BRIN), Bogor, Indonesia; 6 Department of Nutrition and Feed Technology, Faculty of Animal Science, IPB University, Bogor, Indonesia

**Keywords:** bibliometric analysis, international collaboration, methane mitigation, microbiome, rumen fermentation, ruminants, silage feed, sustainability

## Abstract

**Background and Aim:**

Silage plays a pivotal role in ruminant nutrition, significantly influencing rumen fermentation, animal productivity, and environmental sustainability. Despite extensive research on silage and fermentation, a comprehensive synthesis of global trends and collaborations in this domain has not been systematically explored. This study aimed to conduct a bibliometric analysis of global research on silage feed and its effects on rumen fermentation in ruminants. It sought to identify publication trends, leading contributors, research themes, and international collaboration networks, thereby informing future directions in ruminant nutrition research.

**Materials and Methods:**

A total of 1,007 documents published between 1961 and 2024 were retrieved from the Scopus database using targeted keywords. Bibliometric and network analyses were performed using VOSviewer, Bibliometrix (R package), and Microsoft Excel. Inclusion criteria were limited to peer-reviewed English-language articles focused on silage feed and rumen fermentation in ruminants. Data cleaning and preprocessing involved harmonization of author names, keywords, and institutional affiliations.

**Results:**

Publication output has increased significantly since 2010, with China, the United States, and Canada emerging as the top contributors. Major research themes include silage quality, microbial fermentation, methane mitigation, and feed efficiency. Core journals identified include *Journal of Dairy Science* and *Journal of Animal Science*. Leading institutions such as China Agricultural University and the University of Florida demonstrated high productivity and citation impact. Keyword analysis highlighted emerging trends, including microbiome, methanogenesis, and sustainability. Collaboration network analysis revealed strong regional clusters, with North America and Europe forming central hubs, while Asia and South America showed growing but less integrated networks.

**Conclusion:**

Research on silage and rumen fermentation has evolved from foundational studies to interdisciplinary approaches integrating microbiology, environmental science, and precision agriculture. The field is rapidly expanding, with increasing emphasis on reducing methane emissions and enhancing livestock performance through improved silage practices. However, global collaboration remains fragmented, particularly in underrepresented regions. Future research should focus on metagenomics, smart technologies (e.g., Artificial Intelligence and Internet of Things), and policy-driven strategies to optimize feed systems and support sustainable livestock production.

## INTRODUCTION

Silage serves as a cornerstone in ruminant nutrition by providing a reliable, year-round source of preserved forage rich in essential nutrients. The ensiling process is an anaerobic fermentation primarily facilitated by lactic acid bacteria, which convert soluble carbohydrates into organic acids, chiefly lactic acid [1]. This acidification lowers the pH, effectively inhibiting the growth of spoilage microorganisms and preserving the nutritional quality of the forage [2]. Optimizing the conditions of this fermentation process improves silage stability, enhances digestibility, and ensures consistent nutrient availability for ruminant animals.

The significance of silage extends beyond animal performance – it plays a crucial role in promoting sustainable livestock systems. As a feed component, silage supports food security, enhances production efficiency, and helps reduce the livestock sector’s environmental impact, including greenhouse gas emissions. Technological advancements in silage production have focused on improving fermentation efficiency and minimizing nutrient losses. The use of bacterial inoculants, enzymes, and chemical preservatives has proven effective in enhancing both the fermentation profile and aerobic stability of silage, thereby increasing its overall nutritive value [3]. High-quality silage directly contributes to improved feed conversion, better animal productivity, and addresses both economic and ecological challenges in forage-based feeding systems.

The interplay between silage and rumen fermentation is central to ruminant health and environmental performance. Once ingested, silage undergoes microbial fermentation in the rumen, resulting in the production of volatile fatty acids, which serve as the primary energy source for ruminants [4]. The efficiency of this process has a significant impact on livestock metrics, including growth rate, milk yield, and reproductive outcomes [5]. However, suboptimal fermentation – characterized by excessive fiber degradation or poor carbohydrate utilization – can lead to elevated methane emissions, a potent contributor to climate change [6]. By improving silage quality and diet formulation, rumen microbial activity can be optimized, thereby reducing enteric methane emissions and advancing the sustainability of ruminant production systems [7].

Despite substantial progress in understanding silage characteristics and their effects on rumen fermentation, a global synthesis of research activity – encompassing trends, thematic priorities, and collaboration networks – remains lacking. This highlights the importance of bibliometric analysis as a methodological tool for mapping the scientific landscape, revealing research gaps, and guiding future investigations. The present study aims to investigate publication trends, thematic structures, and regional research dynamics within the field of silage and rumen fermentation. By employing bibliometric techniques, this study offers a quantitative assessment of global scientific output. Unlike qualitative reviews, which face challenges in handling large and complex datasets [8], bibliometric analysis enables the systematic evaluation of research development. In the broader context of food and agricultural sciences, bibliometric approaches, alongside systematic reviews and meta-analyses, form a robust framework for evidence synthesis [9–12], and have increasingly been adopted across disciplines to support strategic research planning.

Although the relationship between silage feed and rumen fermentation has been extensively studied from nutritional, physiological, and microbiological perspectives, a comprehensive overview of how global scientific interest in this field has evolved remains absent. Most existing studies focus on experimental evaluations of silage quality, the effects of various feed additives, or methane mitigation strategies in specific ruminant species. However, these investigations are often limited in scope – geographically fragmented, thematically narrow, or lacking in comparative cross-national insights. As a result, there is a limited understanding of the broader research architecture, Including the most influential countries, institutions, authors, and journals driving this domain; how thematic focuses have shifted over time; and where collaboration networks are weak or underdeveloped. Furthermore, as the livestock industry faces increasing pressure to adopt environmentally sustainable practices and smart technologies (e.g., Artificial Intelligence [AI] and Internet of Things [IoT]), there is a critical need to understand whether and how these emerging priorities are being addressed within the academic literature on silage and rumen fermentation. A structured bibliometric analysis is thus essential for identifying underrepresented research areas, tracking thematic evolution, and assessing the alignment of current scientific outputs with global sustainability goals in animal production.

The primary aim of this study is to conduct a bibliometric analysis of global research trends related to silage feed and its impact on rumen fermentation in ruminants, using the Scopus database as the primary source. Specifically, the study seeks to (i) quantify the volume and growth trajectory of scientific publications in this field from 1961 to 2024; (ii) identify leading countries, institutions, authors, and journals contributing to the literature; (iii) map the evolution of research themes, keyword co-occurrences, and emerging scientific interests; and (iv) analyze international collaboration patterns and identify potential gaps in transnational research efforts. By synthesizing this information, the study aims to provide a strategic overview of the intellectual structure and developmental trajectory of silage-related research, highlight areas needing further exploration, and offer evidence-based recommendations for shaping future research agendas that align with sustainable livestock production and global food security priorities.

## MATERIALS AND METHODS

### Ethical approval

As this study was based exclusively on previously published literature and did not involve any human or animal subjects, ethical approval was not required.

### Study period and location

The bibliometric study was conducted between December 2024 and January 2025 at the Animal Husbandry Research Area of the National Research and Innovation Agency, located in Cibinong, Bogor, Indonesia.

### Search strategy and data sources

**Figure 1:**
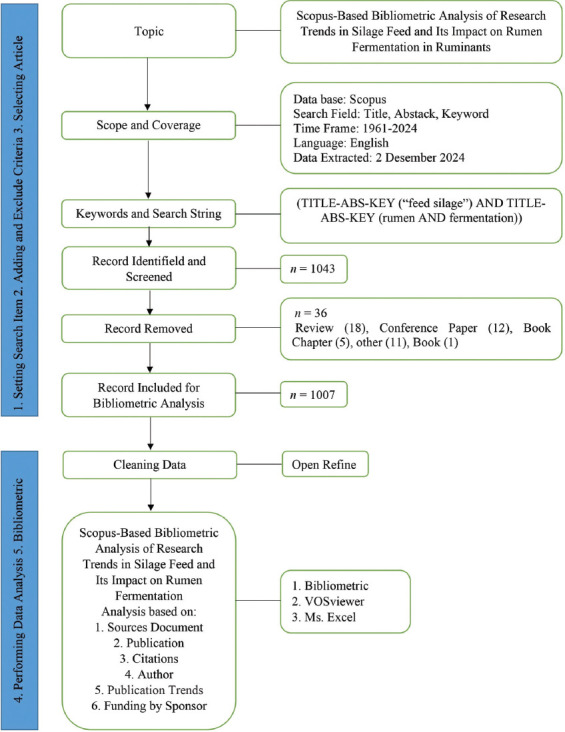
Bibliometric method [13, 14].

This study employed an iterative, structured methodology to identify relevant literature and perform a comprehensive bibliometric analysis. Initial keyword selection was guided by a thorough examination of recent literature on silage feed and rumen fermentation in ruminants, based on theoretical frameworks outlined in prior studies by Fahimnia *et al*. [13] and Fosso Wamba and Mishra [14]. The final search string combined the terms (“Feed” AND “Silage”) with (“Rumen” AND “Fermentation”), ensuring a focused retrieval of publications addressing both feed preservation and digestive processes in ruminants. The literature search was conducted using the Scopus database, covering publications from 1961 to 2024. A six-step workflow was adopted: (1) Formulation of search terms; (2) application of inclusion and exclusion criteria; (3) screening and selection of eligible studies; (4) preliminary data analysis; (5) data cleaning and harmonization; and (6) bibliometric and network visualization (Figure 1) [13, 14].

### Inclusion and exclusion criteria

Inclusion criteria encompassed peer-reviewed journal articles published in English that specifically examined the relationship between silage feed and rumen fermentation in ruminants. Excluded from the analysis were conference proceedings, review papers, book chapters, editorials, non-peer-reviewed publications, and articles unrelated to ruminant species or fermentation mechanisms. The initial search yielded 1,043 records. After applying the defined criteria, 1,007 documents were deemed eligible and included in the final analysis.

### Data extraction and preprocessing

Data extraction was completed on January 10, 2025. The metadata obtained from Scopus included information on authorship, article titles, publication years, institutional affiliations, keywords, funding sources, country of origin, and citation counts. These records were exported in CSV format for further processing. Preprocessing was performed using Refine software and involved three key steps: (1) removal of duplicate entries, (2) standardization of author and institutional names, and (3) normalization of keywords to ensure consistency across datasets.

### Bibliometric and network analysis

The bibliometric evaluation was conducted using VOSviewer (version 1.6.20) [15], the Bibliometrix R package (version 4.2.1) [16], and Microsoft Excel (Office 365). These tools facilitated the analysis of publication volume, keyword co-occurrence, co-authorship networks, institutional linkages, and country-level collaboration patterns. The resulting visualizations and statistics provided a comprehensive overview of dominant research themes, influential contributors, citation distributions, and thematic evolution within the domain of silage and rumen fermentation.

## RESULTS

### Analysis of publication details

**Figure 2:**
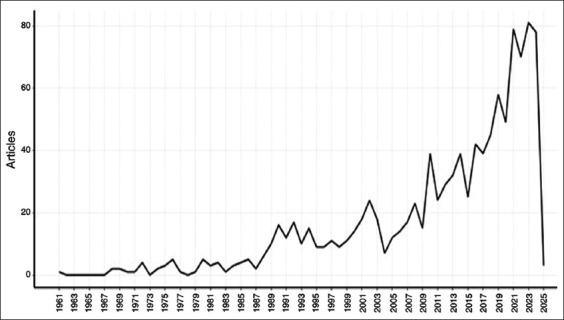
Trends in the number of published documents related to silage feed and rumen fermentation.

Figure 2 and Table 1 present an initial statistical overview of the annual publication trends. Although early research before the 1980s laid the foundational knowledge, the volume of publications remained relatively low and stable. Since the 1990s, however, there has been a noticeable increase in academic interest, which has been particularly accelerated after the early 2000s. A substantial rise occurred around 2010, with a pronounced surge in publication output after 2020, reflecting a clear intensification of research on silage and rumen fermentation.

**Table 1:** General information based on bibliometric data.

No.	Description	Results
1	Timespan	1961–2024
2	Source (Journals, books, etc.)	182
3	Documents	1079
4	Annual growth rate (%)	1.73
5	Average age of documents	13.6
6	Average citation counts per document	26.56
7	References	36287
8	Keywords plus (ID)	2639
9	Author’s keywords (descriptors)	2192
10	Authors	3485

As illustrated in Figure 2 and Table 1, the past decade (2014–2024) has seen a marked increase in publications related to silage feed and its role in rumen fermentation. This trend highlights a growing academic and industrial focus on optimizing silage quality and its impact on ruminant health, productivity, and environmental sustainability [17]. Notably, Table 1 reveals a 14% growth in silage-related research, underscoring the dynamic and evolving nature of this field in recent years.

Contemporary studies have increasingly emphasized precision approaches to silage fermentation, including the use of microbial additives, advanced ensiling techniques, and nutritional profiling to enhance feed efficiency. In parallel, modern research has also explored the broader implications of silage on livestock performance, methane mitigation, and gut microbiota modulation, making this a highly relevant area in current ruminant science.

### Analysis of the number of publications by country

**Figure 3:**
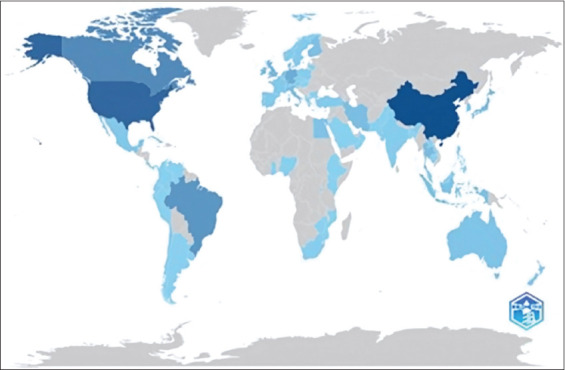
Mapping the number of documents related to silage feed and rumen fermentation by continent.

Table 2 and Figure 3 show the percentage distribution of scientific journals related to animal science by country of origin and the contribution of each journal. The *Journal of Dairy Science*, based in the United States, has the highest number of contributors, totaling 290, which accounts for 21% of the total contributions. Second place was the *Journal of Animal Scienc*e, also from the United States, with 119 (17%). *Animal Feed Science and Technology*, published in the Netherlands, ranks third with 79, representing 13% of the total contribution.

**Table 2:** Top 10 percentage number of documents by country.

No.	Country	No. of documents	%
1	China	1,041	19
2	United States	848	15
3	Canada	550	10
4	Brazil	451	8
5	Germany	252	5
6	United Kingdom	173	3
7	Japan	148	3
8	Thailand	144	3
9	Iran	141	3
10	Ireland	135	2

Other significant journals include *Animals* from Switzerland (32 articles, 8%) and *Animal* from Germany (29 articles, 4%). Germany also contributed 23 articles through the *Journal of Animal Physiology and Animal Nutrition*, with the same percentage (4%). The *Archives of Animal Nutrition* from Japan contributed 22 articles (4%), followed by the *Animal Science Journal* from the Netherlands, which contributed 21 articles (3%). *Livestock Science* from China contributed 18 articles (3%), and the *Chinese Journal of Animal Nutrition*, despite being based in the United States, ranks last with 16 articles (2%).

China has emerged as a leading contributor to silage feed research, driven by substantial innovations across the feed production sector. Improving livestock performance in developing countries requires a multifaceted approach that focuses on innovation, research, and supporting policies. Science, technology, and innovation are crucial for enhancing livestock productivity, marketing, and trade [18]. Supporting researchers and institutions and developing effective national livestock strategies and policies are essential for sustainable development and bridging knowledge gaps in the livestock sector [19].

**Figure 4:**
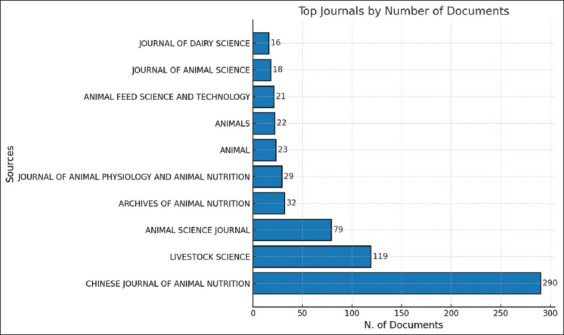
Top 10 article sources about related to silage feed and rumen fermentation.

The distribution of documents related to silage feed and rumen fermentation, as shown in Figure 4 and Table 3, reveals the significant dominance of the United States in the field of animal science. The largest contributions were from the *Journal of Dairy Science* (290 articles, 21%) and the *Journal of Animal Science* (119 articles, 17%). European countries also make important contributions, such as the Netherlands with *Animal Feed Science and Technology* (79 articles, 13%) and *Animal Science Journal* (21 articles, 3%), as well as Germany with *Animal* (29 articles, 4%) and the *Journal of Animal Physiology and Animal Nutrition* (23 articles, 4%). Switzerland further strengthens Europe’s contribution through *Animals* (32 articles, 8%), indicating a focus on animal welfare and sustainability.

**Table 3:** Top 10 percentage number of journals by country.

No.	Journal name	No. of journals	Country of origin	%
1	Journal of Dairy Science	290	United States	21
2	Journal of Animal Science	119	United States	17
3	Animal Feed Science and Technology	79	Netherlands	13
4	Animals	32	Switzerland	8
5	Animal	29	Germany	4
6	Journal of Animal Physiology and Animal Nutrition	23	Germany	4
7	Archives of Animal Nutrition	22	Japan	4
8	Animal Science Journal	21	Netherlands	3
9	Livestock Science	18	China	3
10	Chinese Journal of Animal Nutrition	16	United States	2

Although Asia’s contribution is smaller than that of the United States and Europe, it is beginning to demonstrate its influence. Japan contributed 22 articles through the *Archives of Animal Nutrition* (4%), whereas China, with 18 articles in *Livestock Science* (3%), indicated the rapid growth of research in the region. This reflects the increasing attention to livestock research in Asia, particularly in supporting global food needs. International collaboration in agricultural research, especially in silage feed studies, significantly enhances productivity, sustainability, and innovation [20].

### Core journals and article distribution

**Figure 5:**
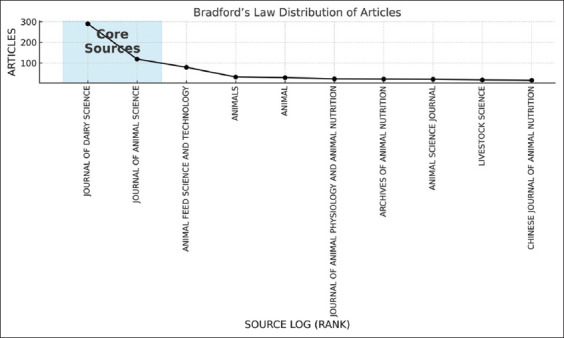
Sources by Bradford’s law.

Figure 5 presents a graph illustrating the distribution of articles based on journal sources, identifying core sources that serve as primary references in research on silage feed and rumen fermentation in ruminants. Journals such as the *Journal of Dairy Science* and the *Journal of Animal Science* dominate the shaded area representing core sources, each contributing over 200 publications. This indicates their critical role in disseminating high-quality research in livestock science, particularly in animal nutrition and rumen fermentation.

The distribution of articles follows a characteristic pattern consistent with Bradford’s Law, where most publications originate from a few core journals, while other journals contribute significantly fewer articles [21]. This trend suggests that researchers prefer internationally recognized journals relevant to their field to disseminate their findings.

The presence of these core sources highlights the importance of access to these journals, both for researchers seeking the latest insights and those intending to publish in this field [22]. Furthermore, the concentration of research within these core journals indicates that silage feed and rumen fermentation are well-established topics in livestock science, supported by a robust body of high-quality and high-impact publications.

### Affiliate analysis by country

**Figure 6:**
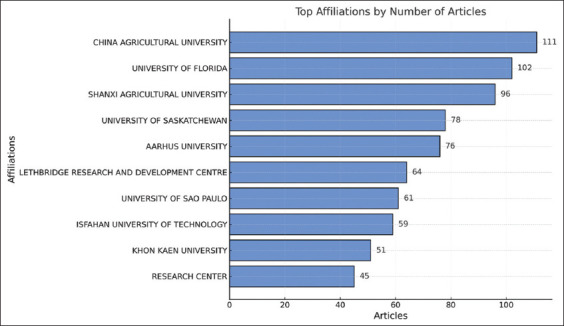
The top 15 affiliations related to silage feed and rumen fermentation.

Figure 6 presents findings highlighting the involvement of various institutions in the research of silage feed and its relationship with rumen fermentation. China Agricultural University has the highest number of articles (111), followed by the University of Florida (102), and Shanxi Agricultural University (96). Significant contributions also come from institutions such as the University of Saskatchewan, Aarhus University, and the University of São Paulo, indicating the active role of various countries in supporting research in this field.

The presence of institutions from regions such as North America, Asia, Europe, and South America reflects the global scope and international collaboration on this topic. The dominance of several institutions suggests that they likely have strong resources, funding, or research focus in this area, making them centers of excellence in research. To expand scientific networks and enhance collaborations, other institutions may consider partnering with these prominent institutions. Additional support from governments or donor organizations to these leading institutions could accelerate future research development.

### Keyword trends and research focus

**Figure 7:**
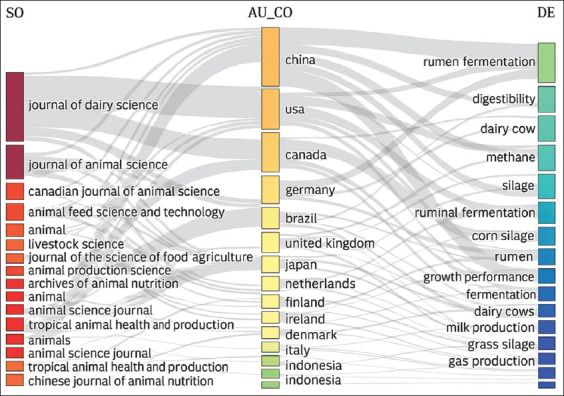
A three-field plot illustrating the source, countries, and keywords related to silage feed and rumen fermentation.

Figure 7 shows that popular research topics include “Rumen Fermentation” and “Digestibility,” reflecting a focus on digestive efficiency and energy production. Topics such as “Methane” and “Silage” highlight attention to environmental impacts and feed management. Research from China and the USA tends to focus on rumen fermentation and digestion, which are aligned with efforts to improve production efficiency and reduce greenhouse gas emissions. Brazil and Canada show interest in “Beef Cattle” and “Corn Silage,” which are relevant to their respective livestock industries.

Global trends show an increased focus on production efficiency and environmental sustainability, reflecting the industry’s need to adapt to challenges such as climate change and food security. This diagram also highlights the potential for international collaboration, where researchers from various countries can share knowledge to achieve common goals. The emphasis on topics such as fermentation and methane emissions may be influenced by government policies that promote more environmentally friendly agricultural practices. Thus, this diagram provides insights into how animal science and production research is adapting to current global challenges.

### Analysis by the author

**Figure 8:**
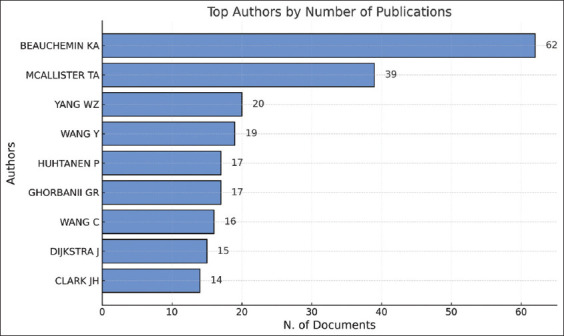
The top 10 authors who researched related to silage feed and rumen fermentation.

The analysis of research on silage feed and its relation to rumen fermentation from 1961 to 2024 reveals significant contributions from various authors. According to Figure 8, the author K. A. Beauchemin is the largest contributor, with 62 published articles, followed by T. A. McAllister (39 articles) and W. Z. Yang (34 articles). Other authors, such as Y. Wang, P. Huhtanen, and G. R. Ghorbani, also made notable contributions, although their document counts were lower than those of the three primary authors.

**Figure 9:**
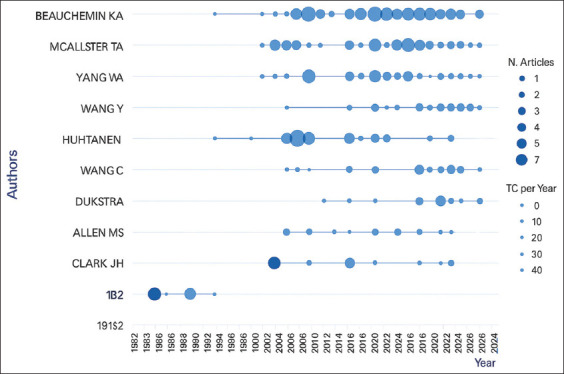
Author productivity related to silage feed and rumen fermentation.

Figure 9 provides further insight into publication trends and the impact of each author’s research. Authors such as K. A. Beauchemin and T. A. McAllister have not only been consistently active in publishing but also have high citation rates, indicating significant influence in the related literature. W. Z. Yang also demonstrated a stable contribution pattern, although their citation rate was slightly lower than those of the other two primary authors. Some authors, such as J. H. Clark, made concentrated contributions in earlier years, which may reflect a shift in research focus or a decrease in publication activity.

### Analysis of the number of citations by country

**Figure 10:**
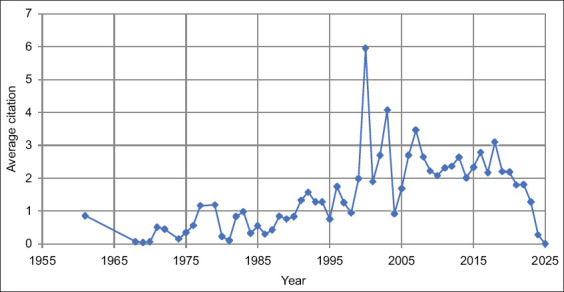
Number of citations about related to silage feed and rumen fermentation.

**Figure 11:**
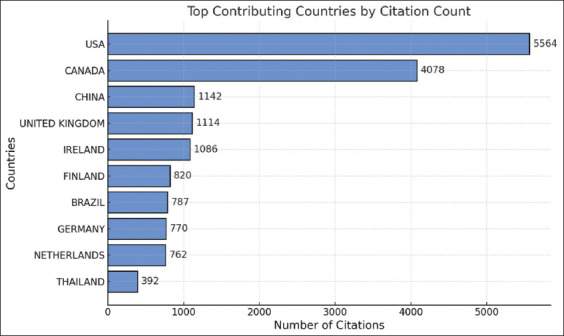
Most cited countries related to silage feed and rumen fermentation 1961–2024.

Figures 10 and 11 and Table 4 illustrates the trend of average citations for silage feed and rumen fermentation research from 1961 to 2024. While citation rates remained low during the early period due to limited studies and technological constraints, a more substantial upward trend became evident starting in the early 2000s. The period from 2000 to 2010 saw a significant increase in citations driven by intensified focus on feed efficiency, the application of microbial inoculants, and advances in analytical and fermentation technologies.

**Table 4:** Top 10 number citations by country about related to silage feed and rumen fermentation.

No.	Country	Total citation	Average article citation
1	USA	5564	49.7
2	Canada	4078	40
3	China	1422	11.3
4	United Kingdom	1412	52.3
5	Ireland	1086	41.8
6	Finland	820	43.2
7	Brazil	787	15.4
8	Germany	770	20.3
9	Netherlands	762	50.8
10	Thailand	392	13.1

Although citation rates remained relatively high after 2010, some fluctuations were observed, with a downward trend emerging around 2015. This decline likely reflects topic saturation and a shift in research priorities. Consequently, the past decade has highlighted the need for innovation in this field. Recent research has increasingly focused on precision additives, modern ensiling technologies and sustainable approaches to maintain the relevance and scientific impact of silage-related studies.

In contrast, Brazil, Germany, the Netherlands, and Thailand report lower total citations, with Brazil and Thailand at the bottom (787 and 392 citations, respectively). However, global trends over the past decade have suggested increasing international collaboration and broader dissemination of knowledge, offering opportunities for improved research quality across various regions.

### Collaboration network analysis by country and keyword identification

**Figure 12:**
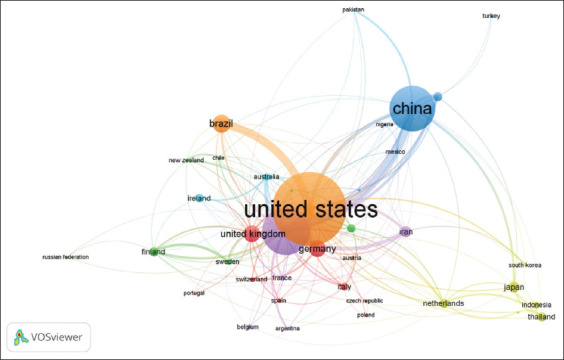
Research conducted jointly between countries on silage feed and rumen fermentation.

Figure 12 displays color-coded clusters of coun-tries based on research collaborations. These clusters reflect shared research themes and citation patterns. The red cluster, comprising the United States, Canada, and Germany, serves as the central hub of this network. Countries within this cluster exhibit strong relationships and frequently collaborate and cite each other, creating a dense and influential network in the context of research.

The blue cluster, which includes China and Turkey, indicates significant connections although not as strong as those in the red cluster. This cluster reflects active collaboration, particularly within the Asian region, highlighting China’s role as a key player in global research.

The green cluster, consisting of Brazil, Argentina, and several South American countries, shows weaker interconnections than the red and blue clusters. Countries within this cluster may engage in more specific research or may be isolated from broader international collaborations.

The yellow cluster, including the Netherlands, the United Kingdom, and other European countries, indicates strong internal relationships among European nations. They exhibit stronger collaboration with each other but may not be directly connected with countries outside Europe.

Finally, the cluster encompassing Indonesia, Thailand, and several Southeast Asian nations appears more isolated, with weaker research connections. These countries may have less involvement in international collaboration than those in other clusters. Overall, the cluster analysis highlights the complex dynamics of global research collaboration, indicating that while some countries have strong networks, others may still be seeking opportunities to engage more deeply in the international research arena.

### Keyword co-occurrence and conceptual mapping

**Figure 13:**
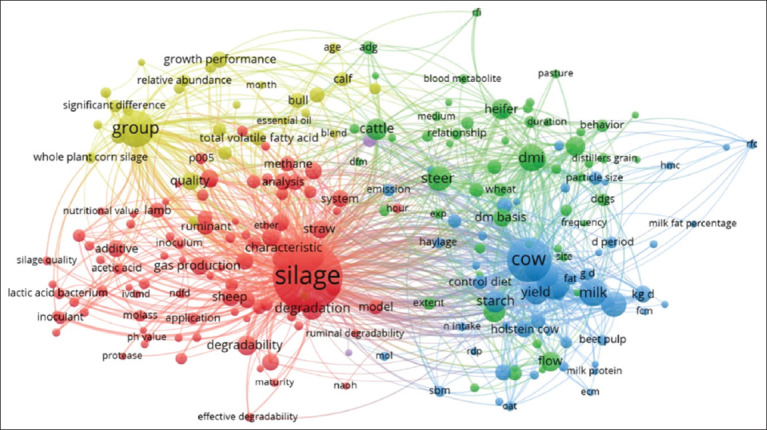
Keyword visualization that often appears in related to silage feed and rumen fermentation.

Figure 13 presents a visual representation of the relationships between various concepts and terms associated with research on silage, animal feed, and animal performance. The color-coded clusters represent groups of interrelated concepts, with the red cluster focusing on silage. Terms such as “silage,” “degradation,” and “characteristic” underscore the significant emphasis on silage quality and how specific factors impact its nutritional value. The strong connections between terms in this cluster reinforce the importance of silage as a central topic in research related to animal feed.

The green cluster centers on cattle and milk production, with terms such as “milk yield” and “starch,” indicating that this research investigates how feed, including silage, impacts milk output. The strong interrelations among these terms underscore the significant focus on optimizing feed to enhance milk production.

The blue cluster includes terms related to feed and intake, such as “dry matter (DM)” and “pasture,” suggesting that this research also examines feed composition and its effects on animal health and performance.

The yellow cluster focuses on group aspects and growth performance, with terms indicating that the research considers how different animal groups may influence research outcomes, as well as significant variations in performance based on certain variables.

Overall, this analysis highlights the complexity and interconnectedness of research in the field of animal feed and nutrition, underscoring the importance of understanding the relationships between silage, feed, and animal performance to optimize outcomes in the livestock industry.

Silage feed serves as a stable, high-quality source of nutrition for cattle, particularly dairy cows, during periods when fresh forage is difficult to obtain. Silage enhances fiber digestibility, provides sufficient energy for milk production, and promotes digestive health in cattle through fermentation, which produces volatile fatty acids essential for metabolism.

### Country-level contributions and international collaboration

**Figure 14:**
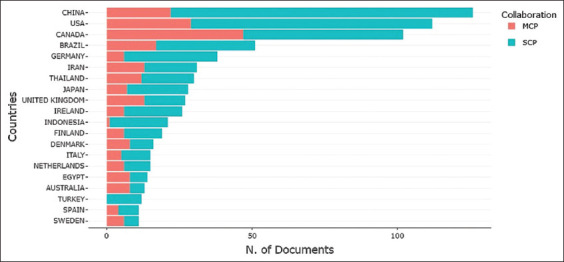
Corresponding author’s countries.

Figures 14 and Table 5 provide information on the top 20 countries based on the number of articles published in the context of research, focusing on single-country publications (SCP) and multinational publications (MCP). China leads with 126 articles, 104 of which are SCP, while 22 are MCP, indicating active involvement in international collaborations. The United States and Canada follow with 112 and 102 articles, respectively, with a balanced proportion of SCP and MCP, signifying that both countries also make significant contributions to global research.

**Table 5:** Top 20 corresponding author countries.

No.	Country	Article	SCP	MCP	No.	Country	Article	SCP	MCP
1	China	126	104	22	11	Indonesia	21	20	1
2	USA	112	83	29	12	Finland	19	13	6
3	Canada	102	55	47	13	Denmark	16	8	8
4	Brazil	51	34	17	14	Italy	15	10	5
5	Germany	38	32	6	15	Netherlands	15	9	6
6	Iran	31	18	13	16	Egypt	14	6	8
7	Thailand	30	18	12	17	Australia	13	5	8
8	Japan	28	21	7	18	Turkey	12	12	0
9	UK	27	14	13	19	Spain	11	7	4
10	Ireland	26	20	6	20	Sweden	11	5	6

SCP=Single country publications, MCP=Multiple country publications, UK=United Kingdom, USA=United States

European countries, such as the United Kingdom, Ireland, and the Scandinavian nations, demonstrate strong contributions, with many publications produced through collaborative efforts. Overall, Table 5 underscores the significance of international collaboration in research and demonstrates how various countries contribute to advancing scientific knowledge.

**Figure 15:**
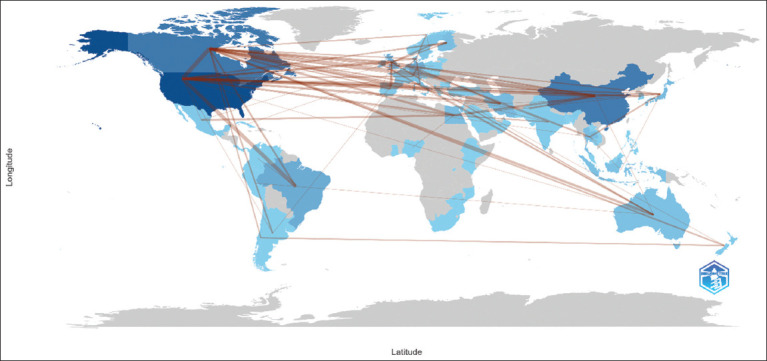
Country collaboration map.

Figure 15 presents a global map illustrating the research collaboration relationships between countries. North America, particularly the United States and Canada, serves as the central hub of collaboration. Europe also displays a strong network of collaboration, whereas Asian countries, such as China and Japan, are involved with lower intensity. In contrast, several countries in Africa and South America appear less connected, reflecting lower engagement in international research. Overall, this map underscores the importance of international collaboration in advancing scientific knowledge and the contributions of various countries to the advancement of global research.

### Analysis of research trends and conceptual structure

**Figure 16:**
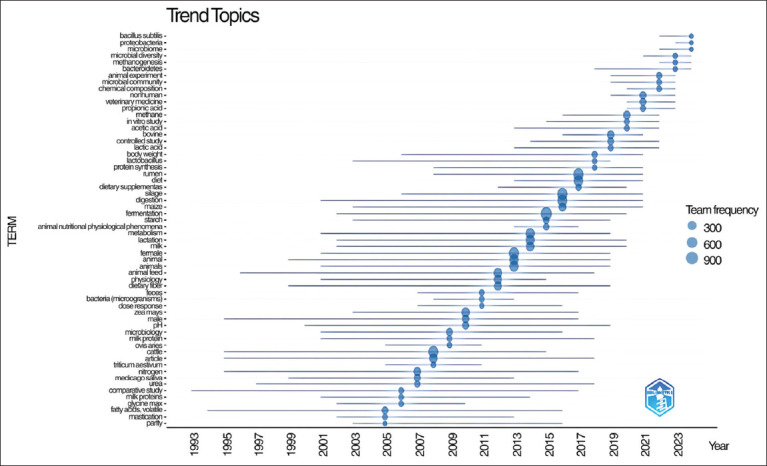
Trending research topics in silage feed and rumen fermentation global.

Figure 16 illustrates the development of major research themes in silage feed and rumen fermentation studies from 1961 to 2024, based on term frequency over time. While earlier decades have shown limited thematic diversity and frequency, a notable expansion of research topics has occurred since the early 2000s. The Y-axis lists key terms representing specific research areas, while the X-axis spans years under study. The size of each circle indicates the relative frequency of a given term, highlighting its prominence in the literature.

In particular, the period from 2000 to 2024 reflects a dynamic shift in research focus, with emerging themes such as microbial additives, fermentation optimization, feed efficiency, and environmental sustainability gaining significant attention. This trend highlights the growing complexity and interdisciplinary nature of contemporary research in this field.

Most of the terms began appearing in significant numbers around 2005 and have continued to increase until 2023. Research topics have evolved over time: Earlier studies were more focused on animal physiology and feed (e.g., dietary fiber, fermentation, and rumen), whereas more recent topics encompass microbiomes and metabolism (e.g., microbiome, Bacteroidetes, and methanogenesis).

### Thematic clusters and evolving priorities

**Figure 17:**
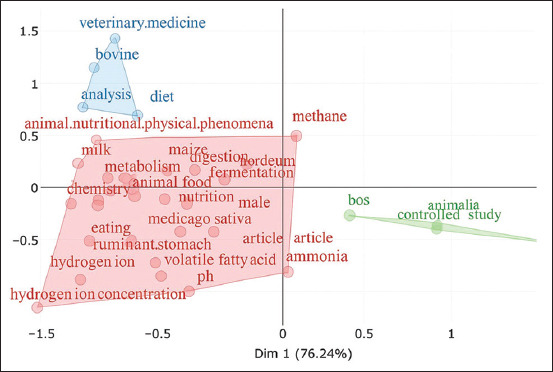
Conceptual structure map of related silage feed and rumen fermentation.

**Figure 18:**
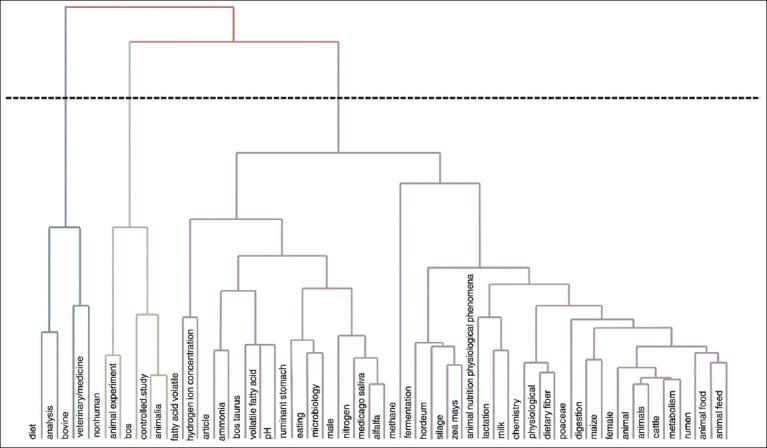
Dendogram factorial analysis.

Figures 17, 18, and Table 6 illustrate key research topics, highlighting fermentation, silage, rumen, cattle, and diet as central themes in the scientific literature. This indicates a strong research focus on rumen fermentation processes and the impact of silage on livestock health and productivity. Keywords such as female, milk, lactation, and dietary fiber reflect an emphasis on animal physiology, particularly milk production, and the effects of diet on female livestock.

**Table 6:** Analysis of research trends related to silage feed and rumen fermentation (1961–2024).

No.	Word	*Dm1	*Dm2	No.	Word	*Dm1	*Dm2
1	Fermentation	−0.25	−0.05	23	Chemistry	−0.82	−0.18
2	Silage	−0.21	0.12	24	Male	−0.25	−0.42
3	Rumen	−0.49	−0.02	25	Bovine	−0.71	1.16
4	Female	−0.68	0.07	26	Eating	−0.76	−0.62
5	Cattle	−0.62	0.06	27	Volatile.fatty.acid	−0.28	−0.88
6	Digestion	−0.4	0.1	28	Medicago.sativa	−0.46	−0.51
7	Diet	−0.57	0.68	29	Ph	−0.33	−1.03
8	Animal	−0.59	0.03	30	Poaceae	−0.42	−0.16
9	Animals	−0.59	0.05	31	Ammonia	0.02	−0.79
10	Metabolism	−0.64	0.03	32	Microbiology	−0.63	−0.63
11	Lactation	−0.68	0.18	33	Non-human	0.97	−0.48
12	Animal.food	−0.54	−0.11	34	Fatty.acids.volatile	−0.8	−0.92
13.	Article	−0.11	−0.73	35	Controlled.study	0.55	−0.37
14	Animal.feed	−0.64	−0.09	36	Veterinary.medicine	−0.65	1.46
15	Zea.mays	−0.29	0.13	37	Hordeum	−0.11	0.05
16	Milk	−0.8	0.25	38	Hydrogen.ion	−0.89	−1.17
17	Physiology	−0.72	−0.23	39	Alfalfa	−0.47	−0.49
18	Ruminant.stomach	−0.64	−0.79	40	Bos	0.29	−0.27
19	Dietary.fiber	−0.73	−0.17	41	Animal.nutritional	−0.72	0.47
20	Maize	−0.49	0.17	42	Analysis	−0.75	0.77
21	Nitrogen	−0.36	−0.47	43	Animalia	0.55	−0.32
22	Methane	0.05	0.47	44	Animal.experiment	1.07	−0.56

* Dm1=Dimension 1; *Dm2=Dimension 2

In addition, terms such as volatile fatty acids, pH, ammonia, and microbiology demonstrate interest in the chemical and microbiological aspects that play a crucial role in rumen fermentation. The study further reveals that research extends beyond biological aspects to practical applications, as indicated by keywords such as controlled study, veterinary medicine, and animal nutrition. Dimensional values (Dm1 and Dm2) provide insights into topic depth and interrelations, with keywords such as “ruminant stomach” and “bovine” indicating subtheme groupings related to ruminant physiology and livestock management.

With this shift in trend, it can be concluded that research in the field of silage feed and its relationship with rumen fermentation is no longer solely focused on traditional aspects, such as livestock diseases, but is also beginning to explore new approaches through microbiology, genetics, and laboratory-based experiments. Researchers interested in this field may consider further exploration of milk microbiology and cattle genetics to enhance production efficiency and animal health.

### Hierarchical keyword clustering

Figure 17 presents a dendrogram that illustrates the hierarchical clustering of keywords in research related to silage feed and rumen fermentation in ruminants. Three main clusters, distinguished by different colors, represent the dominant research themes.

The blue cluster focuses on nutrition and feed formulation aspects, with keywords such as diet, silage, and animal feed, highlighting the critical role of silage in supporting optimal rumen fermentation. The green cluster emphasized controlled studies and the impact of feed on animal health, with keywords such as controlled study, veterinary medicine, and ammonia. Meanwhile, the purple cluster (the largest) encompasses physiological and biochemical aspects, with keywords such as fermentation, rumen, methane, volatile fatty acids, and microbiology. The rumen houses a diverse microbiome, including *Archaea*, Bacteria, Fungi, and *Protozoa*, all of which play crucial roles in the fermentation process [23].

The relationships between keywords are illustrated through connections between nodes, where keywords that are closer together indicate stronger relationships. For example, volatile fatty acids and methane are located in the same branch, indicating a close association between the production of volatile fatty acids and methane emissions during fermentation.

The vertical positioning of the nodes in the dendrogram also reflects the specificity of the relationships, with lower nodes indicating more specific relationships within the cluster. The horizontal lines crossing the dendrogram define the cluster boundaries based on the degree of similarity between the keywords.

## DISCUSSION

### Enhancing silage quality for rumen health

Silage enriched with natural additives such as tannins or probiotics enhances the rumen microbial balance and mitigates the risk of subclinical acidosis caused by excessively rapid carbohydrate fermentation [24]. Silage with an effective fiber content stimulates rumination and saliva production, thereby maintaining optimal rumen pH levels. In terms of productivity, high-quality silage improves livestock weight gain, milk production, and meat quality, particularly in intensive farming systems [25].

### Nutritional composition and performance effects

Mechanically processed alfalfa silage can improve fiber digestibility, feed efficiency, and milk fat content in dairy cows during mid-lactation [26]. Moreover, silage with a higher DM content can reduce lactic acid and ammonia nitrogen levels while increasing water-soluble carbohydrate content [25]. Therkildsen *et al*. [27] have reported that Holstein cattle fed silage show advantages in terms of growth and carcass weight, whereas northern Finncattle demonstrate superior meat flavor quality, although the differences are not substantial.

### Methodological approaches and research priorities

From a methodological perspective, researchers can leverage diverse methodologies and perspectives, improving the quality and applicability of findings in silage feed studies by Samuel *et al*. [28]. In the United States, collaborative meetings between government agencies and dairy organizations have identified research priorities in nutrition, food safety, and sustainability [29].

### Government support and policy frameworks

Government support plays a crucial role in the development and sustainability of the cattle industry. Integrated crop and livestock systems can help reduce deforestation and greenhouse gas emissions from livestock farming [30]. Financial incentives and regulatory frameworks have been shown to have a significant impact on the production and quality standards of meat and milk [31]. Effective government support can be achieved through various strategies, including the digitalization of the industry and the application of modeling techniques to optimize regulatory frameworks [31, 32].

The dominance of the United States in citation counts presents an opportunity for countries worldwide to enhance the impact of their research in this sector, as reported by Nurunnajwa *et al*. [33]. To achieve this goal, governments and research institutions can encourage researchers to publish their work in influential journals by offering appropriate incentives and rewards. Collaborating with researchers from the United States who have demonstrated expertise in the field of silage feed can facilitate knowledge exchange and contribute to research with a global impact.

### Emerging scientific themes and shifts in focus

In recent years, frequently occurring terms include *microbiome*, *Bacteroidetes*, *methanogenesis*, and *microbial diversity*, suggesting a shift in research focus from basic nutrition and physiology to microbial interactions within the rumen and their effects on fermentation and methane production [34]. In addition, there has been a growing trend in research focusing on animal health and nutrition, reflected in the growing use of terms such as *dietary supplements*, *protein synthesis*, *propionic acid*, and *acetic acid*.

Recent studies by Shinkai *et al*. [35] and Li *et al*. [36] have increasingly highlighted the environmental impact of livestock production, as evidenced by the rising occurrence of terms such as *methane* and *nitrogen*, which are associated with efforts to reduce greenhouse gas emissions in livestock systems.

### Keyword mapping and conceptual themes

Figures 17, 18, and Table 6 illustrate key research topics, highlighting *fermentation*, *silage*, *rumen*, *cattle*, and *diet* as central themes in the scientific literature. This indicates a strong research focus on rumen fermentation processes and the impact of silage on livestock health and productivity. Keywords such as *female*, *milk*, *lactation*, and *dietary fiber* reflect an emphasis on animal physiology, particularly milk production, and the effects of diet on female livestock.

Figure 17 presents a dendrogram that illustrates the hierarchical clustering of keywords in research related to silage feed and rumen fermentation in ruminants. Three main clusters, distinguished by different colors, represent the dominant research themes:

- The blue cluster focuses on nutrition and feed formulation, with keywords such as *diet*, *silage*, and *animal feed*, highlighting the critical role of silage in supporting optimal rumen fermentation.
- The green cluster emphasizes controlled studies and the impact of feed on animal health, with keywords such as *controlled study*, *veterinary medicine*, and *ammonia*.
- The purple cluster, the largest, encompasses physiological and biochemical aspects, with terms including *fermentation*, *rumen*, *methane*, *volatile fatty acids*, and *microbiology*. The rumen houses a diverse microbiome, including *Archaea*, Bacteria, Fungi, and *Protozoa*, all of which play crucial roles in the fermentation process [23].

### Role of silage in rumen fermentation and microbiota

Silage plays a crucial role in enhancing rumen fermentation, which is essential for the health and productivity of ruminants. Various types of silage, such as whole-plant corn and *Flemingia* silage, have been shown to positively affect the rumen microbiota [37], fermentation products, and overall animal performance [38].

### Policy and industry implications

The findings of this study have significant implications for stakeholders involved in ruminant nutrition and feed production. Feed producers can leverage these insights to develop advanced silage products enriched with probiotics, enzymes, and organic acids [39, 40], improving feed efficiency and animal health while reducing costs.

Agricultural ministries and policymakers are encouraged to incorporate these research outcomes into sustainable livestock development programs [41, 42] and environmental regulations aimed at mitigating greenhouse gas emissions from ruminant production [43]. By promoting the adoption of innovative silage technologies and real-time monitoring systems, government agencies can support farmers in enhancing feed quality and storage practices, ultimately improving productivity and sustainability [44].

Furthermore, the integration of additives such as tannins and essential oils aligns with global efforts to reduce methane emissions and contribute to climate change mitigation strategies. Collaborative efforts between researchers, industry stakeholders, and policymakers are essential to translate these scientific advancements into practical applications that benefit the entire livestock sector [45].

## CONCLUSION

This bibliometric analysis provides a comprehensive overview of global research trends in silage feed and its impact on rumen fermentation in ruminants. Based on 1,007 peer-reviewed articles retrieved from the Scopus database (1961–2024), the study reveals a sharp increase in publication output over the past decade, particularly after 2010. China, the United States, and Canada emerged as the leading contributors, with strong institutional support from universities such as China Agricultural University and the University of Florida. Core journals, such as *Journal of Dairy Science* and *Journal of Animal Science*, dominated publication sources, highlighting a concentration of impactful research.

Keyword analysis revealed major research clusters centered on fermentation, digestibility, methane mitigation, and microbial dynamics, indicating a shift from conventional nutritional studies toward interdisciplinary approaches that integrate microbiology, environmental science, and sustainability. Recent themes such as microbiome modulation, methanogenesis, and feed efficiency underscore the evolving scientific focus aligned with climate change mitigation goals.

The strengths of this study include the use of a well-established bibliometric framework, advanced visualization tools (VOSviewer and Bibliometrix), and rigorous data cleaning to ensure accurate trend map-ping. The multidimensional analysis, which encompasses country-level productivity, institutional networks, author impact, and thematic evolution, provides a comprehensive view of the research landscape.

However, certain limitations must be acknowledged. The analysis was limited to the Scopus database and English-language articles, potentially overlooking relevant publications indexed in other databases or published in languages other than English. In addition, while bibliometrics quantifies publication patterns and collaborations, it does not assess the methodological quality or scientific validity of the included studies.

Future research should expand bibliometric coverage to include emerging regional databases and gray literature, integrate content analysis for thematic depth, and assess the role of technological innovations (e.g., IoT and AI) in precision silage management. Increased international collaboration, particularly involving underrepresented regions such as Southeast Asia and Africa, is crucial for fostering inclusive knowledge exchange and addressing global food security challenges.

This study demonstrates that research on silage feed and rumen fermentation is rapidly evolving toward more integrated and sustainability-driven directions. Strategic investment in high-quality silage development, rumen microbiota research, and methane-reduction strategies, combined with robust policy frameworks and global collaboration, will be critical to ensuring sustainable ruminant livestock systems in the face of climate and food security challenges.

## AUTHORS’ CONTRIBUTIONS

RM, TRP, WW, and AJ: Data curation, formal analysis, methodology and drafted and revised the manuscript. SA, NQ, DAPS, and TH: Conceptualization. project administration, supervision, and data curation. AE, EW, and YY: Formal analysis. SS, JF, DF, and AEH: Visualization and writing–review and editing of the manuscript. All authors have read and approved the final manuscript.
